# Oral manifestations of dengue viral infection^☆^

**DOI:** 10.1016/j.bjorl.2017.02.006

**Published:** 2017-03-06

**Authors:** Beuy Joob, Viroj Wiwanitkit

**Affiliations:** a Sanitation 1 Medical Academic Center, Bangkok, Thailand; b Joseph Ayobabalola University, Ikeji-Arakeji, Nigeria

Dear Editor,

The recent report on “Oral manifestations of dengue viral infection” is very interesting.^1^ Fernandes et al. noted on “uncommon oral manifestations” in dengue.^1^ In fact, dengue is a highly endemic infection in many tropical countries. In tropical Southeast Asia, this infection is very common. I would like to share the experience from Thailand, a Country with extremely high prevalence on dengue. The oral manifestation of dengue is very common but it is usually mild and can be misdiagnosed by other tropical oro-dental problem.^2^, ^3^ Oro-dental bleeding is a common bleeding presentation in dengue but it is usually not investigated and recorded.^2^ It should be noted that oral manifestation in dengue is not uncommon but usually forgotten by the general practitioner.

## Conflicts of interest

The authors declare no conflicts of interest.
